# Supplementary material for: Scattering spectra models for physics
Source: PNAS Nexus. 2024 Mar 7;3(4):pgae103. doi: 10.1093/pnasnexus/pgae103 (PMC10978061; doi:10.1093/pnasnexus/pgae103)
Supplement: pgae103_Supplementary_Data [file pgae103_supplementary_data.zip › PNASNEXUS-PNASNEXUS-2023-00913R-s02.pdf]

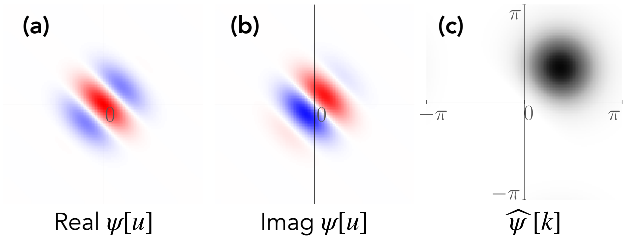

Fig. S1. Real and imaginary parts of a Morlet wavelet $\psi[u]$ and its Fourier transform $\hat{\psi}[k]$, used in numerical calculations.

Supplementary Material

A. Microcanonical Sampling

Given n observed samples $\overline{x}_1, \ldots, \overline{x}_n$ of a field, with possibly $n = 1$, the microcanonical ensemble given in eq. (5) can be extended as follow:

$$\Omega_\epsilon = \Big\{ x_1, \ldots, x_m \in \mathbb{R}^{L^d} \ : \ \|\operatorname*{Ave}_j \Phi(x_j) - \operatorname*{Ave}_i \Phi(\overline{x}_i)\|^2 \leq \epsilon \Big\}. \tag{S1}$$

Microcanonical models are maximum entropy distributions over Ω_ϵ, which have a uniform distribution over this ensemble. Increasing the number of samples n reduces the variance of $\mathrm{Ave}_i \Phi(\overline{x}_i)$ which concentrates around $\mathbb{E}\{\Phi(x)\}$. This reduces the information about a specific realization which is contained in $\mathrm{Ave}_i \Phi(\overline{x}_i)$, thus limiting over-fitting.

Sampling from the microcanonical model amounts to drawing a realization from a uniform distribution in Ω_ϵ. We approximate this sampling with a gradient descent algorithm studied in [1]. This algorithm progressively transports a white Gaussian noise distribution, which has a higher entropy than the microcanonical model, into distributions supported in Ω_ϵ. This is done with a gradient descent on $\ell(y_1, \ldots, y_m) = \|\mathrm{Ave}_j \Phi(y_j) - \mathrm{Ave}_i \Phi(\bar{x}_i)\|^2$, where the y_j are initialized as independent realizations of white noises. At each iteration, the y_i are updated with the L-BFGS-B algorithm, which is a quasi-Newton method that uses an estimate of the Hessian matrix. In practice, we perform 200 gradient descent steps which yield a typical error $\epsilon \approx 10^{-4}$.

It is proved in [1] that this algorithm converges to a distribution that has the same symmetries as $\Phi(x)$, similarly to the microcanonical one. However, it has been shown that this algorithm recovers a maximum entropy distribution in Ω_ϵ only under appropriate conditions and that such gradient descent models may differ, in general, from maximum entropy ones. Nevertheless, these algorithms provide powerful sampling methods to approximate large classes of high-dimensional stationary processes, while being much faster and computationally tractable than alternative MCMC algorithms.

B. Wavelets in $\mathbb{R}^d$ and Scattering Spectra

A Morlet wavelet ψ defined on $\mathbb{R}^d$ is the product of a Gaussian envelope with a sinusoidal wave

$$\psi[u] = g_\sigma[u](e^{i\langle \xi, u\rangle} - c) \ \text{ with } \ g_\sigma[u] = \frac{1}{(\sigma\sqrt{2\pi})^d} e^{-\frac{\|u\|^2}{2\sigma^2}}, \tag{S2}$$

where c is chosen so that $\int \psi[u]\, du = 0$. In practice, the envelope g_σ is an elliptical Gaussian window to increase the angular resolution of ψ, but this does not virtually modify our discussion. Such a wavelet recovers variations around scale 2^j in the direction of ξ. It is invariant to any rotation of $\mathbb{R}^d$ that fixes ξ. In practice we choose $\xi = (3\pi/4, 0, \ldots, 0)$ and $\sigma = 0.8$. For simplifying equations in appendices we assume $\|\xi\| = 1$, without loss of generality. To recover variations at other scales and in other directions we define the wavelet filters

$$\psi_\lambda[u] = 2^{-jd}\psi[2^{-j} r^{-1} u] \ \text{ with } \ \lambda = 2^{-j} r^{-1}\xi$$

for $(j, r) \in \mathbb{R} \times SO(d)$. In Fourier, $\widehat{\psi}_\lambda$ is a Gaussian centered in λ substracted by a Gaussian centered in 0 so that $\widehat{\psi}_\lambda[0] = 0$

$$\widehat{\psi}_\lambda[k] = \widehat{\psi}[2^{-j} r^{-1} k] \ \text{ with } \ \widehat{\psi}[k] = e^{-\frac{\sigma^2}{2}\|k-\xi\|^2} - c\, e^{-\frac{\sigma^2}{2}\|k\|^2}$$

We shall restrict the scales 2^j to dyadic scales, hence taking j integer, and restrict the rotations to a discrete subgroup Γ of $SO(d)$ of order $2^d - 1$ [2]. In dimension $d = 2$ such a group can be parameterized by one angle, in dimension $d = 3$ it can be parameterized by 2 angles. We write $\Lambda = \mathbb{Z} \times \Gamma$ the group that defines filters ψ_λ from ψ.

To guarantee that the wavelet transform W (defined in eq. 9) is invertible and satisfies an energy conservation, we impose that the ψ_λ satisfy the following Littlewood-Paley inequality for $0 < \delta < 1$

$$\forall k \neq 0 \ \ , \ \ 1 - \delta \leq \sum_{\lambda \in \Lambda} |\widehat{\psi}_\lambda[k]|^2 \leq 1 + \delta. \tag{S3}$$

For fields defined on a cubic d-dimensional lattice of length L, the wavelets ψ_λ are discretized accordingly. The wavelet transform is computed up to the largest scale 2^J which is smaller than length L so as to achieve a reasonable estimate of low-frequency moments, even on a single realization. The lower frequencies of x in the ball $|k| \leq 2^J$ are captured by a low-pass filter ψ_0 which is a Gaussian centered in $k = 0$ in Fourier $\widehat{\psi}_0[k] = c_0 \exp(-\sigma_0^2 \|k\|^2/2)$ with $\sigma_0 = \sigma\, 2^{J-1}$. The Littlewood-Paley inequality now reads:

$$\forall k \neq 0 \ \ , \ \ 1 - \delta \leq |\psi_0[k]|^2 + \sum_{|\lambda|^{-1} \leq 2^J} |\widehat{\psi}_\lambda[k]|^2 \leq 1 + \delta. \tag{S4}$$

By applying the Parseval formula we derive that for all x

$$(1 - \delta)\|x\|^2 \leq \|Wx\|^2 \leq (1 + \delta)\|x\|^2 \tag{S5}$$

which insures that W preserves the norm of x, up to a relative error of δ, and is therefore invertible, with stable inverse. For the wavelet used for syntheses of physical fields in this paper, we have $\delta \approx 0.8$.

Covariance of wavelet coefficients $Wx[u, \lambda]$ can be written from the power spectrum $P(x)$ of x

$$\mathbb{E}\{Wx[u, \lambda] Wx[u', \lambda']^*\} = \frac{1}{2\pi} \int P(x)[k] \widehat{\psi}_\lambda[k] \widehat{\psi}_{\lambda'}[k] e^{i\langle u - u', k\rangle} \mathrm{d}k. \tag{S6}$$

It implies that this correlation is zero if the supports of $\widehat{\psi}_\lambda$ and $\widehat{\psi}_{\lambda'}$ do not overlap. For the specified wavelets, as soon as $\lambda \neq \lambda'$, these supports barely overlap and $\mathbb{E}\{Wx[u, \lambda]\, Wx[u', \lambda']^*\} \approx 0$. Moreover, since x is stationary, the covariance $\mathbb{E}\{Wx[u, \lambda]\, Wx[u', \lambda']^*\}$ only depends on $u - u'$ and have a fast decay when the power spectrum $P(x)$ is regular. Thus, even if dependencies across separate scales may exist, they are not captured by correlation.

Taking the modulus of wavelet coefficients removes complex phase oscillations and thus recenter the frequency support of $Wx[u,\lambda]$. Indeed, the power spectrum $P_\lambda(x)$ of $x \star \psi_\lambda$ is mostly supported in a ball $\|k-\lambda\| \leq 2^{-j}\sigma^{-1}$ which does not overlap with the Fourier support of the power spectrum $P_{\lambda'}(x)$ of $x \star \psi_{\lambda'}$. Taking a modulus on $x \star \psi_{\lambda'}$ eliminates the phase which oscillates at the central frequency λ'. As a consequence, the power spectrum of $|x \star \psi_{\lambda'}|$ is centered at $k=0$ and its energy is mostly concentrated in $\|k\| \leq 2^{-j}\sigma^{-1}$ which now may overlap with the support of $P_W(x)[\lambda]$ as can be seen in Fig. 2. The power spectra of $|Wx[u,\lambda]|$ and $|Wx[u,\lambda']|$, both centered at zero, also overlap.

We now justify taking $u = u'$ in order 3 moments given by eq. (13). The cross spectrum $P_{\lambda,\lambda'}(x)$ between $Wx[u,\lambda]$ and $|Wx[u,\lambda']|$ is assumed regular for the fields considered in this paper. In that case one can approximate such cross-spectrum using wavelets, which gives the moments $\mathbb{E}\{WWx[u,\lambda,\gamma]\, W|Wx|[u,\lambda',\gamma]\}$. However, the left-hand-side $WWx[u,\lambda,\gamma]$ is negligible when $\lambda \neq \gamma$ because Fourier support of wavelets ψ_λ and ψ_γ barely overlap. The resulting coefficients

$$\mathbb{E}\{WWx[u,\lambda,\lambda]\, W|Wx|[u,\lambda',\lambda]\} = \frac{1}{2\pi}\int P_{\lambda,\lambda'}(x)[k]|\widehat{\psi}_\lambda|^2 dk \tag{S7}$$

average $P_{\lambda,\lambda'}(x)[k]$ in a ball $\|k\| \leq 2^{-j}\sigma^{-1}$ through $|\widehat{\psi}_\lambda|^2$. However, $P_{\lambda,\lambda'}(x)$ is already concentrated in this ball. We thus remove $|\widehat{\psi}_\lambda|^2$ which yields $\mathbb{E}\{Wx[u,\lambda]\,|Wx|[u,\lambda']\}$.

The following proposition shows that Scattering Spectra reveal non-Gaussianity in a field x.

Proposition 1 *Let x be a stationary process.*

1. *If x is Gaussian then for any separate scales λ, λ', meaning that $\widehat{\psi}_\lambda\widehat{\psi}_{\lambda'} = 0$*

$$\mathbb{E}\{\bar{S}_1(x)\} = \frac{\pi}{4}, \tag{S8}$$

$$\mathbb{E}\{\bar{S}_3(x)[\lambda,\lambda']\} = 0 \quad and \quad \mathbb{E}\{\bar{S}_4(x)[\lambda,\lambda',\gamma]\} = 0. \tag{S9}$$

2. *If x is symmetric i.e. $p(-x) = p(x)$ then*

$$\mathbb{E}\{\bar{S}_3(x)\} = 0. \tag{S10}$$

3. *If x is invariant by rotation of angle π i.e. $p(x[-u]) = p(x[u])$ then*

$$\operatorname{Im}\mathbb{E}\{\bar{S}_3(x)\} = 0 \quad and \quad \operatorname{Im}\mathbb{E}\{\bar{S}_4(x)\} = 0. \tag{S11}$$

Proof If x is Gaussian then $Wx[u,\lambda]$ is also Gaussian and the ratio between its first and second order moment is $\pi/4$. If $\widehat{\psi}_\lambda\widehat{\psi}_{\lambda'} = 0$ then $Wx[u,\lambda]$ and $Wx[u,\lambda']$ are decorrelated, since $(Wx[u,\lambda], Wx[u,\lambda'])$ is Gaussian, this implies that $Wx[u,\lambda]$ and $Wx[u,\lambda']$ are independent. Thus, $Wx[u,\lambda]$ and $|Wx[u,\lambda']|$ are independent, so are $W|Wx|[u,\lambda,\gamma]$ and $W|Wx|[u,\lambda',\gamma]$ which proves 1. Point 2. is proved by observing that $S_3(-x) = S_3(x)$ and point 3. by observing that $S_3(x[-u]) = S_3(x)^*$ and $S_4(x[-u]) = S_4(x)^*$. □

For the physical fields studied in this paper, such coefficients are non-zero, thus revealing their non-Gaussianity Fig.S3.

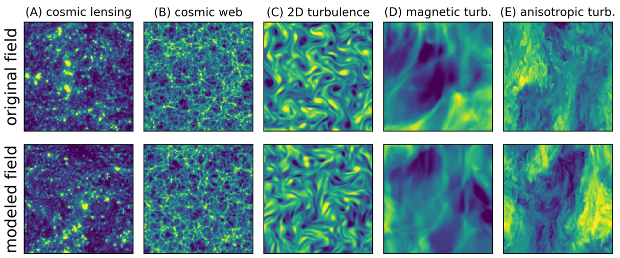

Fig. S2. Visual assessment of our model based on $\bar{S}$ with 11 641 coefficients estimated on a single realization (top). Generated fields (bottom) show very good visual quality.

C. Equivariance and Invariance to rotations and scaling

The scattering spectra are computed from wavelet transforms, which are equivariant to rotations and scalings. We show that scattering spectra inherit these equivariance properties. If $p(x)$ is isotropic or self-similar, then one can build isotropic or self-similar maximum entropy models by averaging renormalized scattering spectra over rotations or scales, which reduces both the variance and dimensionality of $\bar{S}$.

To avoid discretization and boundary issues for rotations and scaling, we consider fields $x[u]$ defined over continuous variables $u \in \mathbb{R}^d$, and establish the mathematical results in this framework. For this purpose, the sum in the wavelet transform defined in eq. (9) is replaced by an integral over $\mathbb{R}^d$. Wavelets are dilated by 2^j for $j \in \mathbb{Z}$ and rotated by r in a rotation group G of cardinal R. In dimension $d = 2$, these rotations have an angle $2\pi\ell/R$.

Proposition 2 *For $r \in G$ with $x_r[u] = x[r^{-1}u]$ one has*

$$S(x_r)[\lambda,\lambda',\gamma] = S(x)[r\lambda, r\lambda', r\gamma]. \tag{S12}$$

For $j \in \mathbb{Z}$ with $x_j[u] = x[2^{-j}u]$ one has

$$S(x_j)[\lambda,\lambda',\gamma] = S(x)[2^j\lambda, 2^j\lambda', 2^j\gamma]. \tag{S13}$$

Proof. It follows from the equivariance of wavelet coefficients, $Wx_r[u,\lambda] = Wx[r^{-1}u, r\lambda]$ and $Wx_j[u,\lambda] = Wx[2^{-j}u, 2^j\lambda]$.

Isotropic fields x have a distribution that is invariant to rotation $x_r \overset{d}{=} x$ for all $r \in G$. Self-similar fields x have a distribution that is invariant to scaling, up to random multiplicative factors $x_j \overset{d}{=} A_j x$ for all $j \geq 0$ [3]. For such fields, we show that the expected scattering spectra exhibit invariance to rotation or scaling of their indices, and thus have a lower-dimensional structure. For that purpose we used normalized scattering spectra coefficient $\bar{S}(x)$ defined eq. (22), where the normalization is done by $\sigma^2[\lambda] = \mathbb{E}\{|Wx[u,\lambda]|^2\}$.

Proposition 3 *If x is isotropic then for any $r \in G$*

$$\mathbb{E}\{\bar{S}(x)[r\lambda, r\lambda', r\gamma]\} = \mathbb{E}\{\bar{S}(x)[\lambda,\lambda',\gamma]\}. \tag{S14}$$

If x is self-similar at scales $2^j \leq 2^J$ then

$$\mathbb{E}\{S_1(x)[\lambda]\} = c_1|\lambda|^{-\zeta_1} \quad , \quad \mathbb{E}\{S_2(x)[\lambda]\} = c_2|\lambda|^{-\zeta_2} \tag{S15}$$

$$\mathbb{E}\{\bar{S}_3(x)[2^j\lambda, 2^j\lambda']\} = \mathbb{E}\{\bar{S}_3(x)[\lambda,\lambda']\} \tag{S16}$$

$$\mathbb{E}\{\bar{S}_4(x)[2^j\lambda, 2^j\lambda', 2^j\gamma]\} = \mathbb{E}\{\bar{S}_4(x)[\lambda,\lambda',\gamma]\} \tag{S17}$$

Proof. Let us assume x is isotropic $x_r \overset{d}{=} x$. It implies that $\mathbb{E}\{S(x_r)\} = \mathbb{E}\{S(x)\}$. Thanks to the equivariance property of (S12) one gets the invariance property on S: $\mathbb{E}\{S(x)[r\lambda, r\lambda', r\gamma]\} = \mathbb{E}\{S(x)[\lambda, \lambda', \gamma]\}$. We obtain (S14) by dividing this equation by $\mathbb{E}\{S_2(x)[r\lambda]\} = \mathbb{E}\{S_2(x)[\lambda]\}$.
Let us assume x is self-similar, $x_j \overset{d}{=} A_j x$. In that case one has $A_{j+j'} \overset{d}{=} A_j A_{j'}$, taking order 1 and order 2 moments, this implies $\mathbb{E}\{A_j\} = 2^{-j\zeta_1}$ and $\mathbb{E}\{A_j^2\} = 2^{-j\zeta_2}$ for certain power-law exponents ζ_1, ζ_2. Now from self-similarity and equivariance property given by (S13) one has $\mathbb{E}\{S_1(x)[2^j\lambda]\} = \mathbb{E}\{A_j\}\mathbb{E}\{S_1(x)[\lambda]\} = 2^{-j\zeta_1}\mathbb{E}\{S_1(x)[\lambda]\}$. Taking $2^{-j} = |\lambda|$ one obtains $\mathbb{E}\{S_1(x)[\lambda]\} = c_1|\lambda|^{-\zeta_1}$ with $c_1 = \mathbb{E}\{S_1(x)[|\lambda|^{-1}\lambda]\}$ independent on $|\lambda|$. With the same reasoning on S_2 we obtain (S15). From self-similarity and equivariance property given (S13), we get similarly: $\mathbb{E}\{S_3(x)[2^j\lambda, 2^j\lambda']\} = 2^{-j\zeta_2}\mathbb{E}\{S_3(x)[\lambda, \lambda']\}$. Dividing by $\mathbb{E}\{S_2(x)[\lambda]\} = c_2|\lambda|^{-\zeta_2}$ yields (S16). We obtain (S17) similarly, which proves the proposition.

The wavelet coefficient renormalization is necessary to ensure that the scattering spectra are invariant to scaling. As explained in [4], it is directly related to Wilson renormalization, which yields macrocanonical parameters (physical couplings) that remain constant across scales (fixed point) at phase transitions, where the field becomes self-similar.

If x is isotropic, then (S14) implies that

$$\operatorname*{Ave}_{r\in G} \bar{S}(x)[r\lambda, r\lambda', r\gamma] \tag{S18}$$

is an unbiased estimator of $\mathbb{E}\{\bar{S}(x)[\lambda, \lambda', \gamma]\}$ with lower variance than $\bar{S}(x)[\lambda, \lambda', \gamma]$. Choosing $\Phi(x) = \operatorname*{Ave}_{r\in G} \bar{S}(x)[r\lambda, r\lambda', r\gamma]$ also reduces the dimension of our model by a factor R. Since this representation is invariant to rotations of x in G, the macrocanonical and microcanonical models defined from it are also invariant to these rotations.

Similarly, if x is self similar on a range of scales $2^j \leq 2^J$, then (S16) and (S17) implies that

$$\operatorname*{Ave}_{j} \bar{S}_3(x)[2^j\lambda, 2^j\lambda'] \quad , \quad \operatorname*{Ave}_{j} \bar{S}_4(x)[2^j\lambda, 2^j\lambda', 2^j\gamma] \tag{S19}$$

where the average is taken on all scales j such that $(2^j|\lambda|)^{-1} \leq 2^J, (2^j|\lambda'|)^{-1} \leq 2^J, (2^j|\gamma|)^{-1} \leq 2^J$, are unbiased estimators of $\mathbb{E}\{\bar{S}_3(x)[\lambda, \lambda']\}$ and $\mathbb{E}\{\bar{S}_4(x)[\lambda, \lambda', \gamma]\}$ with lower variance than $\bar{S}_3(x)$ and $\bar{S}_4(x)$. Choosing $\Phi(x) = (\bar{S}_1(x), \bar{S}_2(x), \operatorname*{Ave}_j \bar{S}_3(x), \operatorname*{Ave}_j \bar{S}_4(x))$ reduces the dimension of our model by at most a factor $\log L$. The resulting maximum entropy model is not necessarily self-similar due to the presence of scale-dependent moments $\mathbb{E}\{\bar{S}_1(x)\}$ and $\mathbb{E}\{\bar{S}_2(x)\}$. However, if $\bar{S}_1(x)[\lambda]$ and $\bar{S}_2(x)[\lambda]$ have a power-law decay along λ our model becomes self-similar.

D. Dimension reduction with Fourier thresholding

We give in this appendix the details of the dimensional reduction of $\bar{S}$ into $P\bar{S}$, which is done by Fourier projectors of $\bar{S}(x)$ along rotations and scales, estimated by thresholding. This dimensional reduction based on regular variations of the dependence of $\bar{S}$ on different scales, allows for a representation of lower variance, bringing the microcanonical and macrocanonical models closer together.

We concentrate on the two-dimensional case $d = 2$ corresponding to numerical applications. The rotation group is then Abelian and defined by a single angle parameter, which simplifies the Fourier transform calculation. However, the same approach applies to non-commutative groups G of rotations in $\mathbb{R}^d$ for $d > 2$, with their Fourier transform. Each wavelet frequency is defined in eq. (8) by $\lambda = 2^{-j} r_\ell \xi$, where r_ℓ is a rotation of angle $2\pi\ell/R$. To guaranty that the scattering spectra frequencies satisfy $|\lambda| \leq |\lambda'| < |\gamma|$, we write

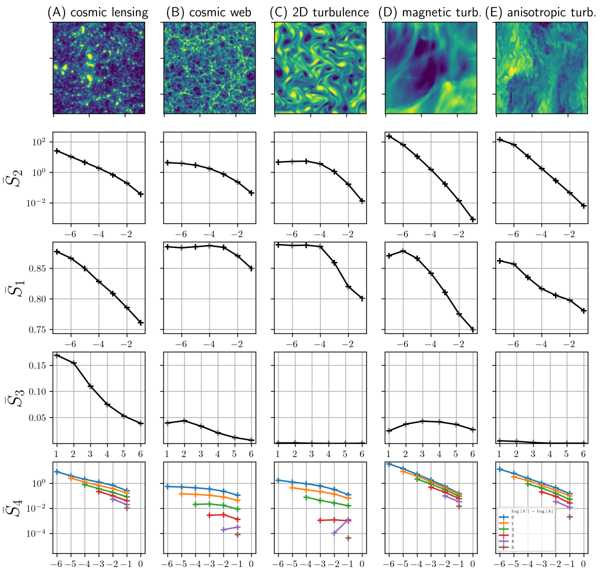

Fig. S3. Visualization of Scattering Spectra $\bar{S}$ for different physical fields. Power-spectrum S_2 and sparsity factors $\bar{S}_1$ are averaged along all angles (amount to taking the 0-th angle Fourier harmonic). We only show the 0-th angle Fourier harmonic and 0-th scale Fourier harmonic for order 3 and order 4-moment estimators $\bar{S}_3$ and $\bar{S}_4$. Thus, the quantities that are shown are invariant to the rotation of the field, and the last two rows ($\bar{S}_3, \bar{S}_4$ are furthermore invariant to scaling). Non-zero coefficients $\bar{S}_3$ show that the cosmic lensing and cosmic web fields are not invariant to sign flip. This is due to the presence of high positive peaks on the former and filaments on the latter. The large amplitude of envelope coefficients $\bar{S}_4$ on the last 2 fields indicate long-range spatial dependencies as evidenced by the presence of structures at the level of the map.

$$\lambda = 2^{-j_1} r_{\ell_1}\xi \ , \ \lambda' = 2^{-j_1-a} r_{\ell_2}\xi \ , \ \gamma = 2^{-j_1-b} r_{\ell_3}\xi \tag{S20}$$

with $0 \leq a < b \leq J - j_1$ and $J < \log L$. It leads to a scale and angle reparametrization of the scattering spectra:

$$\bar{S}(x)[\lambda, \lambda', \gamma] = \bar{S}(x)[j_1, a, b, \ell_1, \ell_2, \ell_3]. \tag{S21}$$

If $\bar{S}(x)$ has regular variations as a function of rotations then its three-dimensional Fourier transform along the (ℓ_1, ℓ_2, ℓ_3) has coefficients of negligible amplitude at high frequencies, which can thus be eliminated. One can also take advantage of regularities along scales. Since $1 \leq j_1 \leq J$ varies on an interval without periodicity, the Fourier transform is replaced by a cosine transform along j_1 for a and b fixed. We could also perform a cosine transform along the scale shift a and b, but this is not done in numerical applications because their range of variations is small and j-dependent. The Fourier transforms along j_1 is however sufficient to identify scale-invariance, since one then expects $\bar{S}$ to only depend on a and b, see appendix 4. We write $F\,\bar{S}(x)$ the Fourier transform of $\bar{S}(x)$ along (ℓ_1, ℓ_2, ℓ_3) and its cosine transform along j_1.

Since F is unitary, it preserves the estimator variance:

$$\sigma^2_{\bar{S}} = \mathbb{E}\{\|\operatorname*{Ave}_i F\bar{S}(x_i) - \mathbb{E}\{F\bar{S}(x)\}\|^2\}. \tag{S22}$$

Ideally, the estimation error of $\mathbb{E}\{F\bar{S}(x)\}$ is reduced by eliminating its coefficients whose squared amplitude is smaller than the variance of the empirical estimation error. It amounts to suppressing all coefficients having a variance that is larger than the bias resulting from their elimination. However, we can not implement this optimal "oracle" decision because we do not know $\mathbb{E}\{F\bar{S}(x)\}$. In this paper, we instead apply an approximate thresholding algorithm, which eliminates small amplitude coefficients of $\bar{S}(x)$ below a threshold proportional to their standard deviation, as discussed in the main text. This thresholding algorithm is adaptive and the selected coefficients vary from one process to another. For each process studied, an ensemble of between 20 to 100 samples $\{x_i\}$ were used to empirically estimate the average and variance of $F\bar{S}$, called $\mu(F\bar{S})$ and $\sigma(F\bar{S})$. The coefficients which have been kept are those that individually verify $\mu(F\bar{S}) > 2\sigma(F\bar{S})$.

A projected scattering spectra

$$\Phi(x) = P\,\bar{S}(x) \tag{S23}$$

is computed with a linear Fourier projection P which eliminates all coefficients of $F\bar{S}(x)$ corresponding to coefficients of $\mathrm{Ave}_i F\bar{S}(x_i)$ below their threshold. The efficiency of this projected scattering is the variance reduction ratio $\sigma^2_{P\bar{S}}/\sigma^2_{\bar{S}}$ with

$$\sigma^2_{P\bar{S}} = \mathbb{E}\{\|\operatorname*{Ave}_i P\bar{S}(x_i) - \mathbb{E}\{P\bar{S}(x)\}\|^2\}. \tag{S24}$$

If $p(x)$ is isotropic or self-similar then we expect that P is a low-frequency projector along global rotations (which act similarly on all l_i coordinates) or scalings (which act on j), which corresponds to the averages described in (S18) and (S19). The Fourier projection P is however much more general and can adapt to unknown regularities of $p(x)$ along rotations and scales.

E. Number of coefficients for shell binned polyspectra

For a 2D field, there are originally $O(L^4)$ bispectrum coefficients in total, as there are two independent frequencies in the bispectrum and each has two dimensions. If we take N_{bin} linear frequency bins along each side of L lattice points, the coefficients to be estimated is reduced to $O(N^4_{\mathrm{bin}})$. A rotation and parity average will further reduce and better estimate the bispectrum coefficients, which eliminates one dimension and leads to $\sim \frac{1}{A^3_3}\cdot\frac{1}{2}N_{\mathrm{bin}}\cdot\frac{3}{4}N^2_{\mathrm{bin}}\cdot\frac{1}{2} = \frac{1}{32}N^3_{\mathrm{bin}}$ binned coefficients, where $1/A^3_3 = \frac{1}{6}$ is the repeated counting of the three-frequency combinations in bispectrum, $\frac{1}{2}N_{\mathrm{bin}}$ is the number of choice of k_1, given rotation invariance, $\frac{3}{4}$ is the number of choice of k_2 given the requirement that each k is within the $L\times L$ lattice in Fourier space and $k_1+k_2+k_3=0$, and the factor $\frac{1}{2}$ comes from parity average.

For 2D fields, the shell-binned bispectrum is essentially a fast way to compute the rotation and parity average of the bispectrum. It does not mix very different configurations, because a given set of $|k_1|, |k_2|, |k_3|$ combined with the condition $k_1+k_2+k_3=0$ uniquely set the configuration up to free rotations. The number of coefficients is of the order $\sim \frac{1}{8}N^3_{\mathrm{bin}}$ (the scaling power is 3 rather than $2d = 4$ because the orientation average eliminates one degree of freedom). For our choice of $N_{\mathrm{bin}} = 10$, there are 151 shell-binned bispectrum coefficients. Similarly, the shell-binned trispectrum $\bar{T}$ has $651 \sim \frac{1}{16}N^4_{\mathrm{bin}}$ coefficients. Note that the shell-binning for trispectrum is more aggressive, because in 2D the same set of $|k_1|, |k_2|, |k_3|, |k_4|$ may come from different combinations k_1, k_2, k_3, k_4 even if the condition $k_1+k_2+k_3+k_4=0$ is applied.

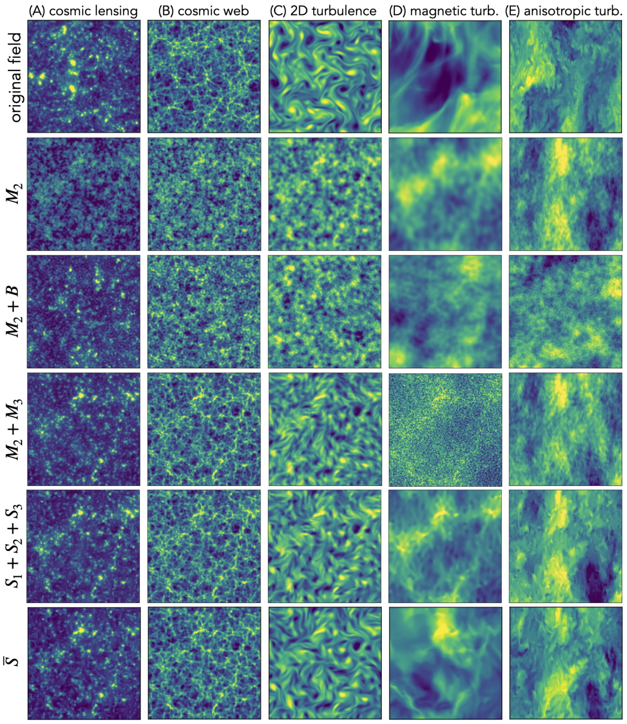

Fig. S4. The improvement of modeled fields from using only the power spectrum and bispectrum to the scattering spectra.

The ordering of $\bar{B}$ and $\bar{T}$ shown in Fig. 4 is determined in a nested way. The frequency annuli are labeled by i from small to large $|k|$. To remove redundant coefficients, we require $i_1 \le i_2 \le i_3 (\le i_4)$ and order them first by i_1 in increasing order; when two binning configurations have the same i_1, they are then ordered by i_2 and so on.

F. Improvement from power spectrum to scattering spectra

In Figure 3 we have shown the modeled fields with up to 4th order moments and scattering spectra. Here we demonstrate step by step the improvement from the traditionally used power spectrum to the powerful $\bar{S}$ in Figure S4. The power spectrum (2nd row) is clearly not able to reproduce any non-Gaussian structures, it is able to capture the strong anisotropy in field E as we measure it with 4 different orientations. The next row shows results with isotropic bispectrum defined as eq. (26). We modified the $|k|$ bins to be logarithmically spaced because a linear binning causes a convergence issue of the algorithm. The sparse peaks in field A start to emerge, but characteristic structures in other fields such as filaments, swirls, and stream lines are still missing. The fourth row demonstrates the improvement from replacing the bispectrum by the selected 3rd order wavelet moments M_3, with which richer structures emerges in field B and C. There is a convergence issue for field D which demonstrates the numerical instability of higher-order moments. The last two rows then show the improvements from replacing higher-order moments to scattering spectra, up to 3rd and 4th order.

G. Logarithm in field B

In our main text we have built the scattering spectra model on the logarithm of cosmic density field (field B) to characterize the cosmic web structure. An interesting question arises as whether the model performs equally well without the logarithm, where the fluctuations becomes dominated by a few high peaks and the cosmic web structure is overshone by the peaks in terms of amplitude. We have performed experiments to explore this regime. We find the scattering spectra model can reproduce many peaks and the bright filament structures, but much worse for the highest peaks and faint filaments than on the field with logarithm transform. In addition, although it can reproduce a reasonably well fit skewed PDF with a fast drop on the left and long tail on the right, it cannot completely forbid negative values; the very few highest peaks are also not bright enough. Given that the morphology of a web requires good characterization of the voids which have density close to zero, it is no wonder that our model does not well reproduce the faint filaments. For comparison, although the cosmic lensing field (field A) is a projected cosmic density field and also has a log-normal PDF, it is much less skewed due to the physical projection along the line of sight. As a result, although we do also observe some deviation of higher-order moments between the model and original fields caused by the log-normal PDF, it is not as severe a problem as the field B before logarithm. Also, the much flatter decrease on the low end of PDF in field A reflects the fact that cosmic web structures are averaged out due to projection.

We find that the failure of modeling field B without logarithm can be fixed by either imposing the logarithm as we have done in the main text, which guarantees the cutoff at zero and makes whole PDF more Gaussian [e.g., 5], or by adding coefficients to our model which explicitly constrain the PDF profile in a differentiable manner, using e.g., smoothed histogram [4] or the mean values of different quantiles. However, the convergence time with the latter solution is increased by a factor of ten and requires balance between the loss function from the PDF and scattering constraints. A thorough study on incorporating the PDF constraints to the scattering spectra model is left for future work.

References

1. Joan Bruna and Stéphane Mallat. Multiscale sparse microcanonical models. *Mathematical Statistics and Learning*, 1(3):257–315, 2019.
2. Y. Meyer. *Wavelets and Operators*. Advanced mathematics. Cambridge university press, 1992.
3. Benoit B Mandelbrot, Adlai J Fisher, and Laurent E Calvet. A multifractal model of asset returns. *Cowles Foundation discussion paper*, page 1412, 1997.
4. Tanguy Marchand, Misaki Ozawa, Giulio Biroli, and Stéphane Mallat. Multiscale data-driven energy estimation and generation. *Phys. Rev. X*, 13:041038, Nov 2023.
5. Mark C. Neyrinck, István Szapudi, and Alexander S. Szalay. Rejuvenating the Matter Power Spectrum: Restoring Information with a Logarithmic Density Mapping. *"Astrophysical Journal Letter"*, 698(2):L90–L93, June 2009.
