# Supplementary figures and images for: Scattering spectra models for physics
Source: PNAS Nexus. 2024 Mar 7;3(4):pgae103. doi: 10.1093/pnasnexus/pgae103 (PMC10978061; doi:10.1093/pnasnexus/pgae103)

(a)

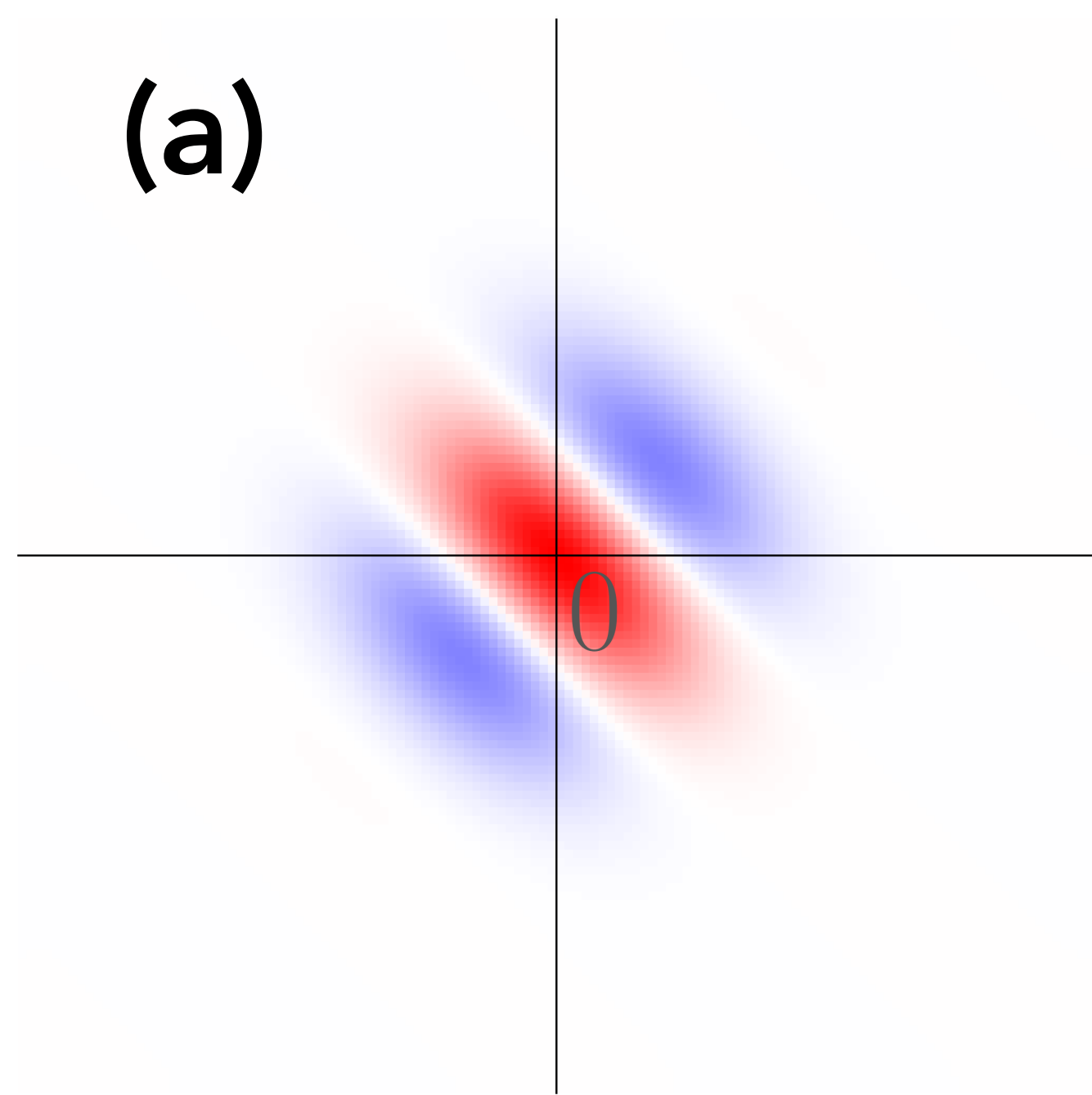

$\text{Real } \psi[u]$

(b)

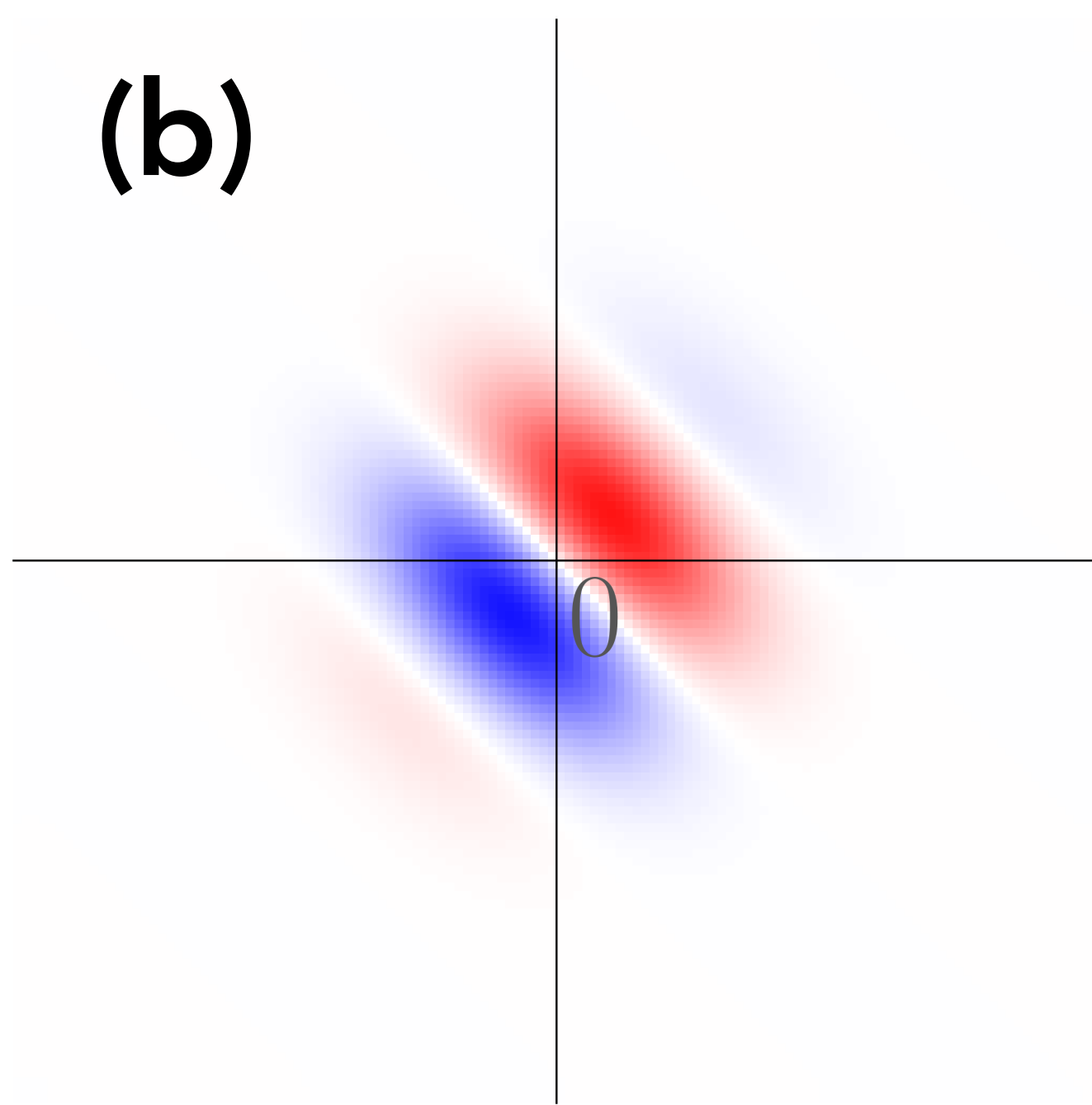

$\text{Imag } \psi[u]$

(c)

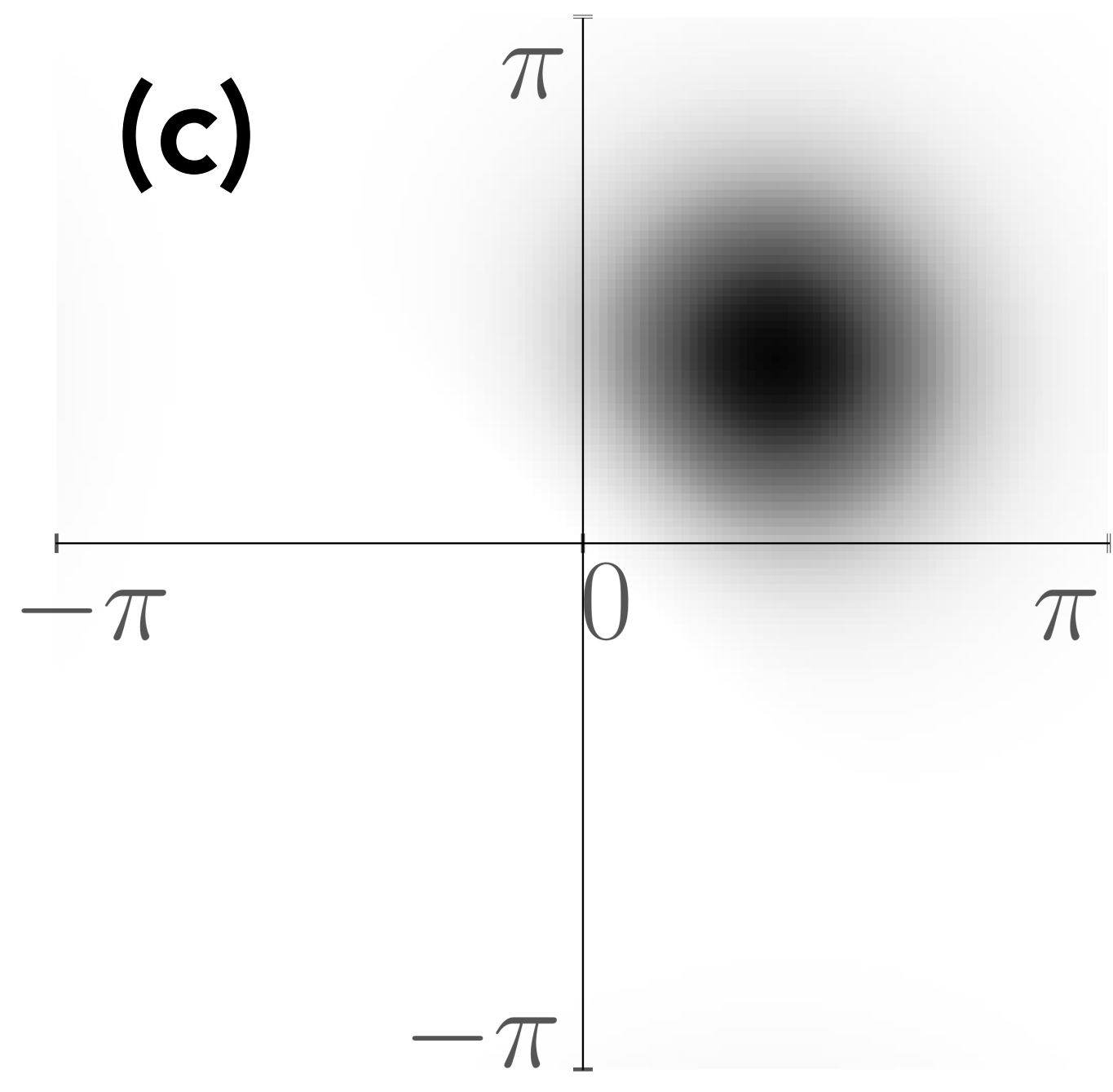

$\hat{\psi}[k]$

Supplement: pgae103_Supplementary_Data [file pgae103_supplementary_data.zip › PNASNEXUS-PNASNEXUS-2023-00913R-s01.pdf]

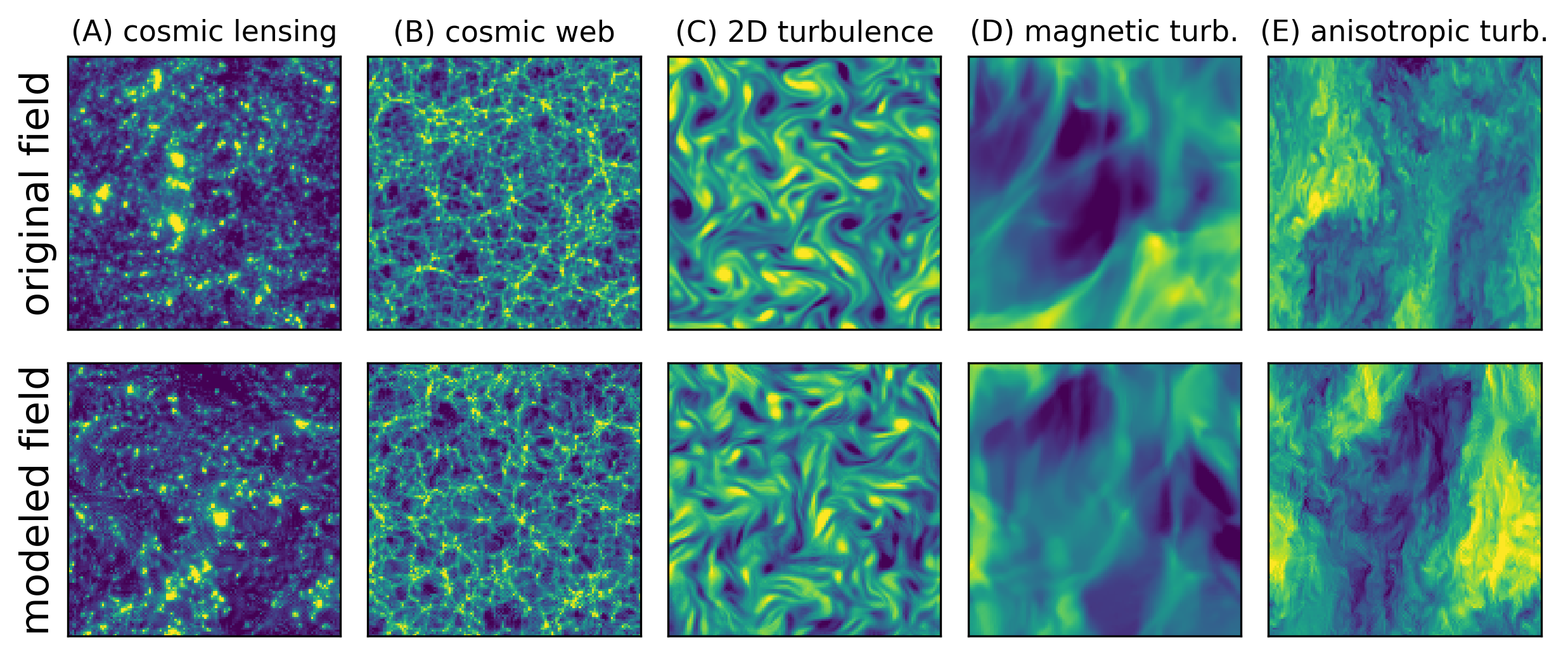

Supplement: pgae103_Supplementary_Data [file pgae103_supplementary_data.zip › PNASNEXUS-PNASNEXUS-2023-00913R-s03.png]

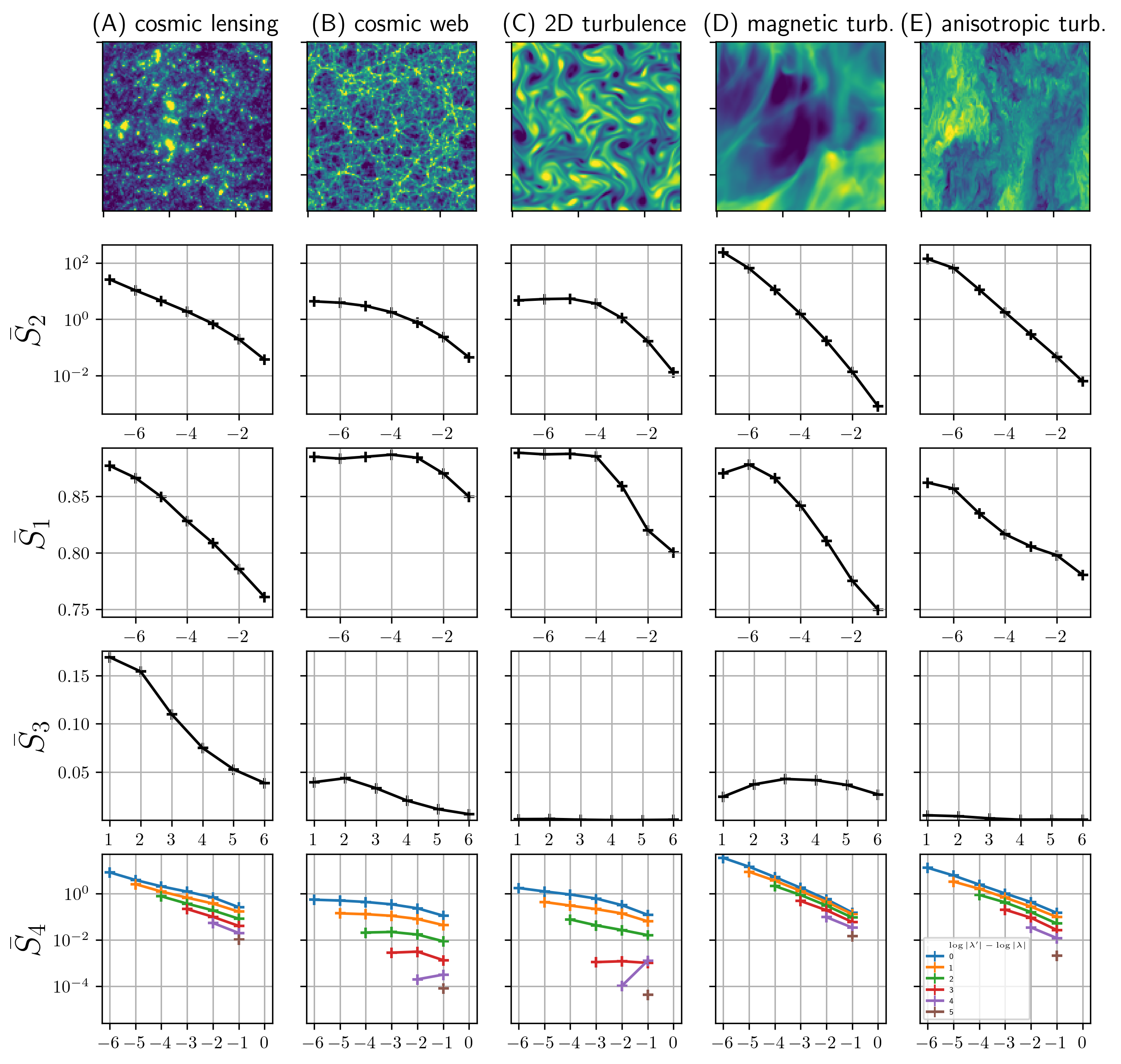

Supplement: pgae103_Supplementary_Data [file pgae103_supplementary_data.zip › PNASNEXUS-PNASNEXUS-2023-00913R-s04.png]

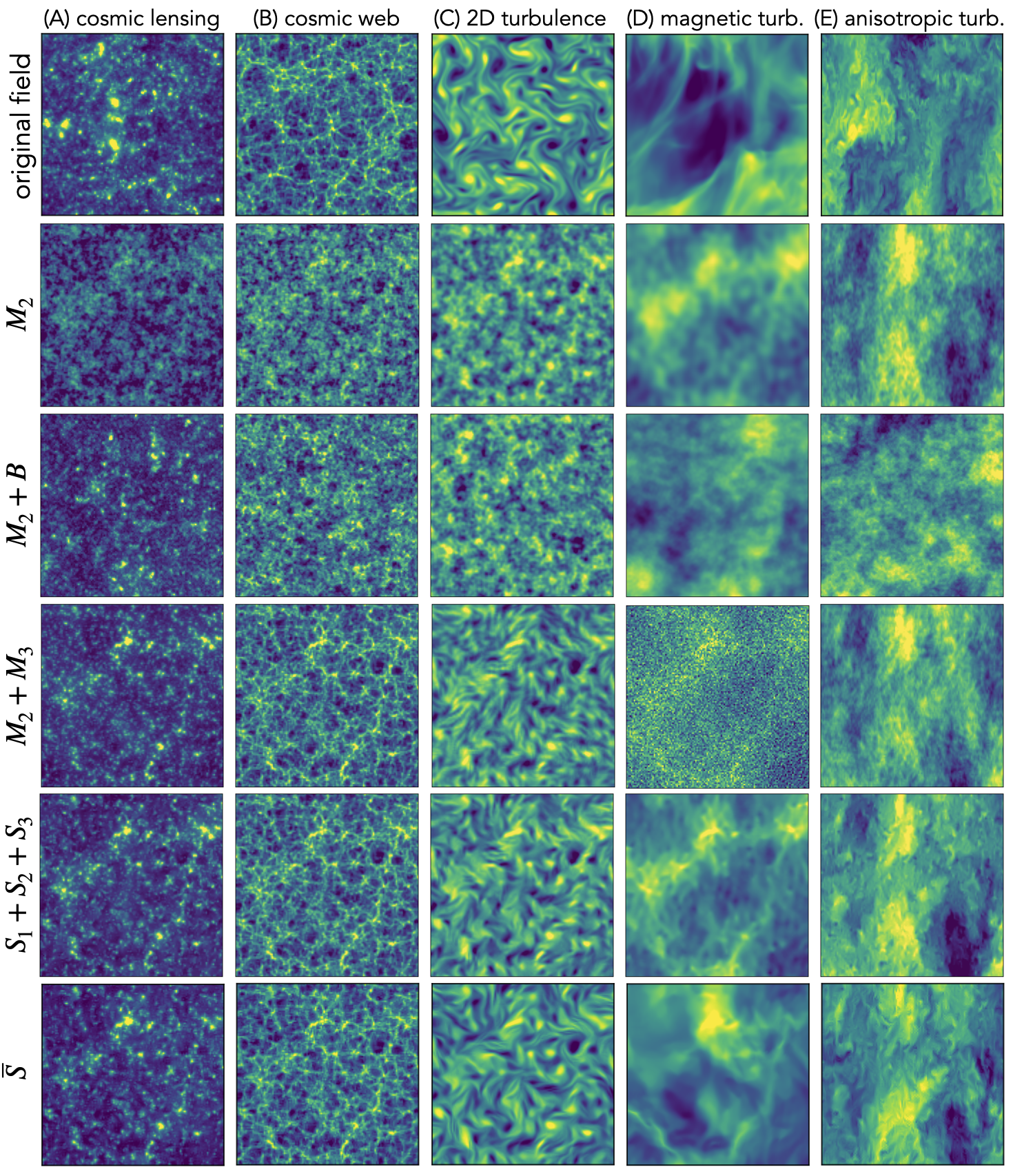

Supplement: pgae103_Supplementary_Data [file pgae103_supplementary_data.zip › PNASNEXUS-PNASNEXUS-2023-00913R-s05.png]
